# A Water Solution from the Seeds, Seedlings and Young Plants of the Corn Cockle (*Agrostemma githago*) Showed Plant-Growth Regulator Efficiency

**DOI:** 10.3390/plants14152349

**Published:** 2025-07-30

**Authors:** Jana Ambrožič-Dolinšek, Vid Golič, Víctor Rouco Saco, Petra Peranić, Veno Jaša Grujić, Terezija Ciringer

**Affiliations:** 1 Department of Biology, Faculty of Natural Sciences and Mathematics, University of Maribor, Koroška 160, SI-2000 Maribor, Slovenia; vid.golic@student.um.si (V.G.); vroucosaco@gmail.com (V.R.S.); petra.peranic1@um.si (P.P.); veno.grujic@um.si (V.J.G.); terezija.ciringer@um.si (T.C.); 2 Department of Elementary Education, Faculty of Education, University of Maribor, Koroška 160, SI-2000 Maribor, Slovenia; 3 Faculty of Biology, Universidade de Santiago de Compostela, Rúa Lope Gómez de Marzoa, s/n, 15782 Santiago de Compostela, Spain

**Keywords:** *Agrostemma*, plant growth regulators, PGRs, cytokinin, auxin, *Triticum* bioassay, mung bean bioassay, *Cucumis* bioassay, Agrostemin^®^, biostimulants, bioassay

## Abstract

Corn cockle (*Agrostemma githago* L. (*Lychnis githago* (L.) Scop.)) is the main ingredient in some plant preparations for biostimulation in agriculture, and it elicits many positive responses. In our study, we attempted to determine if the fresh and dry plant material of *A. githago* contained auxin-like and cytokinin-like growth regulators (PGRs). *Cucumis* and mung bean bioassays were used to determine the presence of auxin-like PGRs and *Cucumis* and *Triticum* bioassays were used to determine the presence of cytokinin-like PGRs. A water solution derived from the crushed, homogenized and extracted seeds, fresh and dry seedlings, and fresh and dry young plants showed auxin-like activity in both bioassays. The activity in the *Cucumis* bioassay corresponded to 0.5 to 2 mg L^−1^ of Indole-3-butyric acid (IBA), and in the mung bean bioassay, the activity corresponded to 0.5 to 4 mg L^−1^ of IBA. While the same water solutions showed weak or no cytokinin-like activity in the *Cucumis* cotyledon expansion bioassay, and they showed an activity of approximately 0.5 to 1 mg L^−1^ of 6-Benzylaminopurine (BAP) in the *Triticum* bioassay. An LC-MS analysis confirmed the presence of free auxins, low levels of or no auxin analogues, a small amount of free cytokinins and a higher level of their cytokinin analogues in the samples, seeds, dry seedlings and young plants of *A. githago,* which was likely related to the fine-tuning between the free and analogue forms of the PGRs in the water solutions used in the experiments.

## 1. Introduction

Agricultural cultivation practices have evolved towards ecological, sustainable and environmentally friendly systems. Natural plant biostimulants and plant conditioners that stimulate natural processes in plants have been proposed as innovative solutions for sustainable agriculture and have received considerable attention from both the scientific community and commercial companies, especially in the last two and a half decades, but also earlier [1,2]. Plant biostimulants (PBS) are described as materials containing substances and/or microorganisms whose function, when applied to plants or rhizospheres, is to stimulate natural processes to improve and/or prefer nutrient uptake, nutrient efficiency and tolerance for abiotic stress and/or crop quality, regardless of their nutrient contents [3]. The formulations of biostimulants for plants are generally proprietary compositions based on algal extracts, complex organic materials, plant-hormone-like compounds, amino acids and humic acids [4]. The composition and complexity of different biostimulants are partly unknown, and sometimes it is even difficult to understand what the most active compounds are. For this reason, the classification of biostimulants is usually based on their effects on or physiological responses of the plants and not on their composition [5]. Effective biostimulants can be connected to phytohormones, key factors in the regulation of plant growth and development [6].

*Agrostemma githago* L. (synonym *Lychnis githago* (L.) Scop.) or the common corn cockle (also written as “corncockle”), Caryophyllaceae, is a herbaceous annual flowering plant. It grows up to 100 cm tall and has lanceolate, opposite (paired) leaves. It used to be a common weed in cereal fields in the hinterlands and in southern Europe, but today, it is less common because of the use of herbicides and mechanized agriculture since wheat is harvested before the seeds can develop [7]. A mutualistic relationship between *A. githago* and cereals has been demonstrated, but this depends on population density. When grown together, both components make better use of the mineral nutrients in the substrate [8]. *Agrostemma githago* is also known as a poisonous plant. Its seeds have been the focus of chemical analyses since the 19th Century, with publications about the isolation of “agrostemmin,” likely a mixture of saponins and proteins grouped into two groups of bioactive components, and ribosome-inactivating proteins (RIPs) and triterpenoid saponins, also called agrostemmosides [7]. The genome of this species, attractive for its phytochemical and pharmacological applications, has been analyzed [9].

Agrostemin^®^ [10] is a preparation developed in the 1980s that is described by its producer as a bioenergent, bioregulator, natural fertilizer, bioactivator, positive allelopathic substance and a preparation used to improve plant productivity. Today, this type of product is called a biostimulant [1]. It is derived from higher plant sources and can be called a “higher plant-derived biostimu-lant” (hPDB) [11]. The chemical analysis of Agrostemin^®^ [10] has shown that it consists of two groups of plant origin-organic compounds. It is a mixture of free amino acids (tryptophan, glutamic acid and orcialanin) and their derivatives, other amino acids, organic acids and their derivatives (allantoic acid, cordianin and adenine). It also contains inhibitors in trace amounts such as ABA (abscisic acid) derivates, and saturated aliphatic hydrocarbons and cyclic inhibitor in trace amount with the structure C_8_H_29_N_3_O_7_. The formulation is a mixture of 4% of the active complex and 96% of the neutral carrier substance Mg_2_SiO_4_ [10].

Corn cockle (*Agrostemma gitago* L. (*Lychnis githago* (L.) Scop.)) is the main ingredient in the plant preparation Agrostemin^®^ [10]. In our previous work, we demonstrated that the biostimulant Agrostemin^®^ [10] elicits a specific physiological response that has a growth-regulating effect and exhibits specific plant-hormone-like properties [12]. Therefore, the next step of the study was to investigate whether the plant source, *Agrostemma githago* L. or common corn cockle, also exhibits plant-hormone-like properties, more precisely auxin-like or/and cytokinin-like activity at different growth stages. Furthermore, the investigation would also consider the extent to which this activity could be comparable to that of the synthetic plant growth regulators (PGRs) commonly used in plant biotechnology. The aim of our study was to investigate the cytokinin and auxin effects of the seeds, seedlings and young plants of *A. githago* at different stages of the life cycle, using bioassays to compare them with those of Agrostemin^®^.

## 2. Results

**Figure 1:**
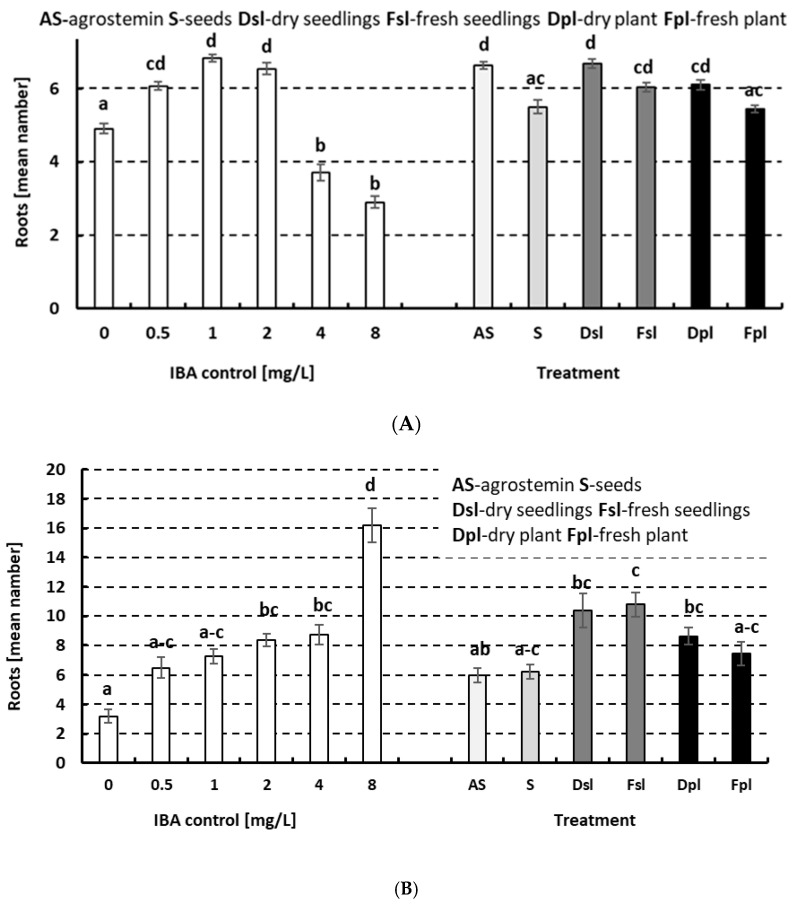
(**A**) The auxin-like activity detected in *Agrostema githago* using a *Cucumis* cotyledon root formation bioassay and (**B**) a mung bean root formation bioassay. A comparison of the effects of IBA control solutions to plant material and Agrostemin^®^ water solutions. Water solutions from the seeds (S), dry and fresh 5-day-old seedlings (Dsl and Fsl), and dry and fresh 10-day-old plants (Dpl and Fpl) of *A. githago* in 500 mg L^−1^ were compared to the recommended dose of water extract (500 mg L^−1^) of Agrostemin^®^. The mean root number (*n* = 40) and SE are presented. The results were statistically evaluated using the Kruskal–Wallis test, followed by post hoc comparisons. Significant differences are indicated by the different letters (a–d).

The water extracts of the seeds, dry and fresh 5-day-old seedlings, and dry and fresh 10-day-old plants of *A. githago* in amounts of 500 mg L^−1^ were compared to the recommended dose of water extract (500 mg L^−1^) of Agrostemin^®^. The seeds, dry and fresh seedlings, and fresh and dry plants of *A. githago* and Agrostemin^®^ showed auxin-like activity in a *Cucumis* cotyledon root formation bioassay and a mung bean root formation bioassay compared to their butyric acid (IBA) equivalents (Figure 1A,B). This effect was observed in all materials tested, including the control Agrostemin^®^ at the recommended concentration. The activity was less pronounced in seeds than in the other preparations. The effect was more pronounced in seedlings and in young plants. Notably, the effect was more pronounced in dry material than in fresh material. The activity in the cucumber bioassay corresponded to approximately 0.5 to 2 mg L^−1^ of IBA and was not higher. Higher IBA concentrations significantly suppressed cucumber root formation. The activity in the mung bean bioassay corresponded to 0.5 to approximately 4 (or even higher) mg L^−1^. Interestingly, higher IBA concentrations were even beneficial to and not suppressive of mung bean root formation.

An LC-MS analysis confirmed the presence of auxins in all the samples, seeds, dry seedlings and dry plants of *A. githago* including Agrostemin^®^ (Table 1). The highest amount was found in the seedlings. Smaller amounts were found in the young plants and seeds, and the smallest amount was found in the biostimulant Agrostemin^®^. The analogues Indole-3-acetic acid (IAA) with aspartic acid and tryptophan (IAA-Asp, IAA-Trp) were detected only in the seeds and the analogue Indole-3-acetic acid (IAA) with aspartic acid (IAA-Asp) only in the seedlings.

**Figure 2:**
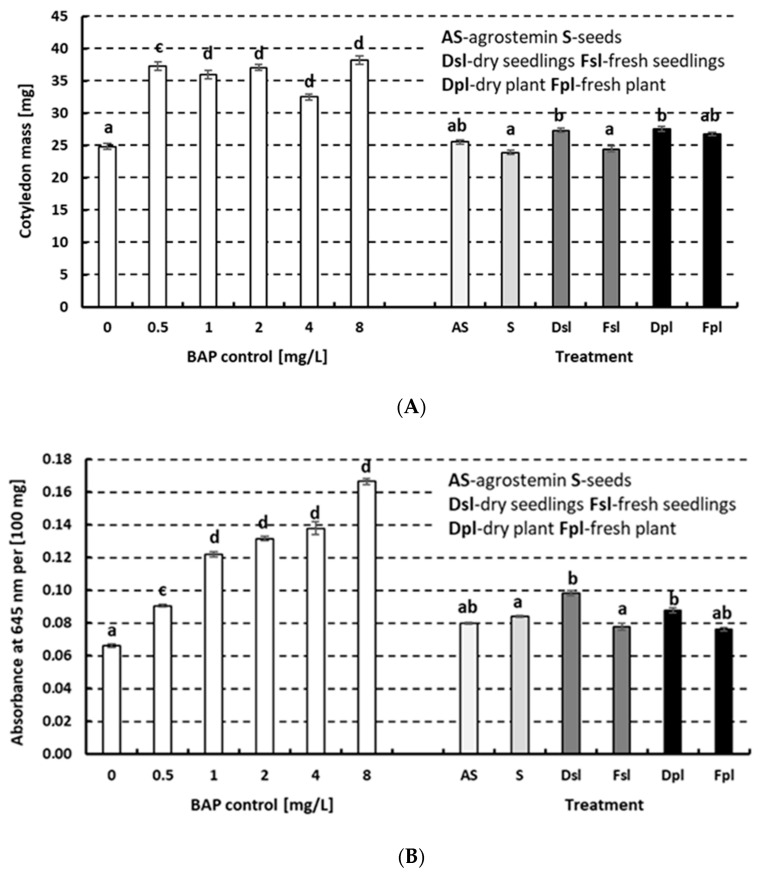
The cytokinin-like activity detected in *Agrostemma githago* using (**A**) a *Cucumis* cotyledon expansion bioassay and (**B**) a *Triticum* bioassay. A comparison of the effect of the BAP control solutions to the plant material solutions and Agrostemin^®^ water solutions. Water solutions of the seeds (S), dry and fresh 5-day-old seedlings (Dsl and Fsl), and dry and fresh 10-day-old plants (Dpl and Fpl) of *A. githago* at 500 mg L^−1^ were compared to the recommended dose of a water extract (500 mg L^−1^) of Agrostemin^®^. (**A**) The mean FW of 10 cotyledons (*n* = 40) and SE are presented. (**B**) The mean chlorophyll absorbance of 10 leaf segments (*n* = 40) and SE are presented. The results were statistically evaluated using the Kruskal–Wallis test, followed by post hoc comparisons. Significant differences are indicated by the different letters (a–d).

The cytokinin-like activity of Agrostemin^®^ was determined with the *Cucumis* and *Triticum* tests. The seeds, fresh and dry seedlings, and fresh and dry plants of *A. githago* and Agrostemin^®^ did not show any cytokinin-like activity when compared to 6-Benzylaminopurine (BAP) (Figure 2A,B) when the *Cucumis* test was used. However, the *Triticum* monocots test also showed some cytokinin-like activity. The activity was significantly less pronounced in the plant material than in the control. Notably, the effect was more pronounced in the dry material than in the fresh material. The effect corresponded to approximately 0.5 to 1 mg L^−1^ of BAP.

An LC-MS analysis confirmed the presence of cytokinins in all the samples of the seeds, dry seedlings and dry plants of *A. githago* including Agrostemin^®^ (Table 2). The highest amount was found in young plants. Slightly smaller amounts were found in seedlings, smaller in seeds, and the smallest amount was found in the biostimulant Agrostemin^®^. The amounts of analogues c-ZeatinRiboside (c-ZR) and t-ZeatinRiboside (tZR) detected in seedlings and young plants exceeds the amounts of free forms of c-Zeatin (cZ) and t-Zeatin (tZ). The amounts of analogue forms detected in seeds were lower than free forms. Agrostemin^®^ did not contain detected amounts of t-Zeatin (tZ) and t-ZeatinRiboside (t-ZR). 

The LC-MS analysis also confirmed the presence of other PGRs such as cis-trans abscisic acid (ABA), salicylic acid (SA) and the oxylipins jasmonic acid (JA), jasmonic acid-isoleucine (JA-Ile) and 12-oxo-phytodienoic acid (OPDA) (Table 3).

## 3. Discussion

*Agrostemma githago* (corn cockle) is an example of a biostimulant derived from higher plant sources and is summarized by the phrase “higher plant-derived biostimulant” (hPDB) [11]. *Agrostemma githago* (corn cockle) stimulates natural plant processes, controls growth and development, and contains phytohormone-like substances, among other bioactive compounds [11,12]. There are other hPDBs, for example, the leaf extract from *Moringa oleifera,* which increases the amount of auxins, gibberellins and cytokinins in *Phaseolus vulgaris*. This promotion is most likely due to the presence of the phytohormones, auxins, gibberellins and cytokinins in *M. oleifera* [13]. Other well-known examples of such stimulating effects are willow extract (*Salix* sp.), young corn (*Zea mais*) and coconut water (*Cocos nucifera*). They are mostly used in research or organic farming. Their phytohormone concentrations are usually not strictly standardized, and this is their known limitation [13].

Bioassays are essential components in the investigation of biological activity [14]. Bioassays are small-scale tests that assess the biological activity of a substance by evaluating their effects on living model organisms [15]. Root growth assays are old and widely used to evaluate the regulatory potential of plant compounds during growth, differentiation [15] and even stress responses. They are simple, sensitive and inexpensive detection methods that can be used to detect and estimate PGR-like activities in PDBs, providing insights into their biological activity through observable physiological responses. In our previous work, we showed that the biostimulant Agrostemin^®^ [10] elicits a specific physiological response that has a growth-regulating effect and exhibits plant-hormone-like properties [12].

The main ingredient of the plant preparation Agrostemin^®^ is *A. githago*, and thus, the logical decision was to test whether the corn cockle exhibits plant-growth regulators and cytokinin- or/and auxin-like activity at different growth stages by comparing it with the synthetic plant growth regulators (PGRs) commonly used in plant biotechnology. Bioassays that are carried out mainly with agronomically important monocotyledonous cereals have already shown that extracts from the seeds of *A. githago* stimulate the growth of young wheat plants [16]. These results were consistent with the allelopathic effect of *A. githago* on wheat seedlings, biomass and grain production obtained in a growth chamber experiment in pots [8] and in wheat under aseptic conditions [17].

Our study has confirmed that *A. githago* water extract contains and shows growth regulators and cytokinin- and/or auxin-like activity at different growth stages in fresh or dry samples, and it was comparable to synthetic analogues. This effect was even more pronounced than the effect of the biostimulant Agrostemin^®^. The auxin-like substances in *A. githago* have been shown to initiate root formation. In both tests—the mung test and the *Cucumis* test—initiation may have been the result of a synergistic effect of the wounding and auxin. Wounding initiates the formation of sink tissue for the transport of assimilates to the sinks for wound healing and cell division, while auxins stimulate the differentiation of the vascular cambium and the development of adventitious roots [18]. This is the possible reason why the formulation based on *A. githago* could replace synthetic auxins in the rooting of a cutting. The stimulatory role of *A. githago* in rooting may be useful in organic and sustainable agriculture, especially since synthetic plant growth regulators are not recommended in organic farming [19] and can be replaced by naturally produced ones [20].

Once the active formulations are identified, a chemical analysis should be performed for more precise identification and quantification of the PGRs in the *A. githago* plant material used for water suspensions. Our LC-MS analysis confirmed the presence of high levels of free auxins and low levels of or no auxin analogues in the samples from the seeds, fresh and dry seedlings, and fresh and dry young plants of *A. githago*, including Agrostemin^®^. Our LC-MS analysis confirmed the presence of small amounts of the free cytokinins c-zeatine and t-zeatine (c-Z and t-Z, respectively) and higher levels of their cytokinin analogues in most of the samples of the seeds, dry seedlings and dry and young plants of *A. githago*. The preparation of Agrostemin^®^ was devoid of cytokinin analogues. This suggests that the fine-tuning between the conjugated and non-conjugated forms of the auxins and cytokinins may have altered the growth and development, as well as the response, of the plant material to the *A. githago* water extract in the bioassays.

Our LC-MS analysis also confirmed the presence of other PGRs, such as cis-trans-abscisic acid, salicylic acid, jasmonic acid (JA), jasmonic acid-isoleucine and 12-oxo-phytodienoic acid, which may also modulate plant growth and development and deserve further bioassays and analyses. The next step should be to analyze the roles of the other PGRs analyzed in our experimental system, which can also be performed using bioassays. In addition, further studies would allow us to better understand how the water extracts of *A. githago* modulate phytohormone balance or the overlap of phytohormones. This would enable us to understand the signaling pathways that affect important physiological and molecular processes [21].

## 4. Materials and Methods

### 4.1. Plant Material

Water solutions from the seeds, freeze-dried and fresh 5-day-old seedlings, and freeze-dried and fresh 10-day-old plants of *A. githago*, called young plants, were used to investigate their cytokinin and auxin effects using bioassays, and we compared these to a water solution of Agrostemin^®^ [10]. The freeze-dried and fresh plant materials were crushed, homogenized and extracted for two hours in an appropriate volume of deionized water. The supernatant of the water solution, extracted from the plant material, was used in experiments. The same procedure was used for the preparation of Agrostemin^®^ with *Agrostemma githago* as the main active constituent [10]. Water solutions from the seeds, seedlings and plants of *A. githago* at 500 mg L^−1^ were compared to the recommended dose of 500 mg L^−1^ of a water solution of Agrostemin^®^. The recommended dose of Agrostemin^®^ [10] was 500 mg in 1L of water (500 mg L^−1^).

### 4.2. Biological Activity

For testing the auxin-like activity of the plant materials, we used the cucumber cotyledon root formation bioassay developed by Zhao and colleagues [22] and the mung bean root formation bioassay described by Yopp [23]. Both assays were adapted, modified and described in detail by Ambrožič-Dolinšek and colleagues [11].

The excised cucumber cotyledon root formation bioassay [22] was prepared as follows: cucumber seeds, “Kumare Dolga zelena” Semenarna Ljubljana [24] and with an expected germination time of 10–14 days, which were germinated in the dark. Forty cotyledons were used for each treatment. These cotyledons, excised from the 10-day-old cucumber seedlings, were placed in four Petri dishes on a filter paper disk and treated with auxin 3 mL indole-butyric acid (IBA) (0, 0.5, 1, 2, 4 and 8 mg L^−1^), plant extracts or Agrostemin^®^ water solutions, all of which were dissolved in deionized water. The cotyledons were incubated in the dark at 25 ± 2 °C for 7 days to determine the auxin-like activity. Deionized water was used as the negative control. The number of roots was counted and compared with the activity of the IBA control. Each treatment was replicated four times, and the experiment was repeated three times.

The mung bean root formation bioassay [23] was prepared as follows: mung bean (*Vigna radiata* L. Wilczek.) seeds were washed, soaked and sown into vermiculite. The seeds were then planted in moistened vermiculite in plastic trays and placed at 25 °C in a light/dark period (16/8 h) with a light intensity of 30–60 μmol m^−2^ s^−1^ for approximately 7–10 days and regularly watered. For the bioassay, 16 uniform seedling segments with carefully removed cotyledons were used with 6–7-cm-long hypocotyls cut 3 cm below the cotyledons. Four seedling cuttings were immersed for 24 h in glass vials containing 10 mL of indole-butyric acid (IBA) (0, 0.5, 1, 2, 4 and 8 mg L^−1^), plant extracts or Agrostemin^®^ water solutions, all of which were dissolved in deionized water. Deionized water was used as the negative control. The vials were returned to the original growth conditions for 7 days. The solution was regularly replenished with distilled water to the original level. The number of adventitious roots longer than 1 mm were counted at each hypocotyl after 7 days of growth and compared with the activity of the IBA control. Each treatment was replicated four times, and the experiment was repeated three times.

For testing the cytokinin-like activity of the plant materials, we used the excised cucumber cotyledon expansion bioassay developed by Zhao and colleagues [22] and the *Triticum* (wheat) leaf chlorophyll retention test developed by Kühnle and colleagues [25]. Both bioassays were adapted, modified and described in detail by Ambrožič-Dolinšek and colleagues [11].

The excised *Cucumis* cotyledon expansion bioassay [22] was prepared as follows: cotyledons for the bioassay were prepared as in the root development test described above. Forty cotyledons were used for each treatment. These cotyledons, cut from the 10-day-old seedlings, were placed on a disk of filter paper in 4 6 cm Petri dishes and treated with 3 mL of cytokinin-6-benzile-amino-purine (BAP) (0, 0.5, 1, 2, 4 and 8 mg L^−1^), plant extracts or Agrostemin^®^, all of which were dissolved in deionized water. The cotyledons were incubated in the dark at 25 ± 2 °C for 4 days to determine the cytokinin activity. Deionized water was used as the negative control. The fresh weights of 10 cotyledons were recorded and compared with the activity of the different BAP concentrations. Each treatment was replicated four times, and the experiment was repeated three times.

The *Triticum* (wheat) leaf chlorophyll retention test [25] was prepared as follows: the seeds of the wheat varieties (*Triticum aestivum* L.) were rinsed under running tap water for 24 h. They were planted at a depth of 1 cm in moistened vermiculite in plastic trays and placed in a growth chamber at 25 °C in a light/dark period (16/8 h) for approximately 7–10 days. The seeds were watered regularly. The fully expanded leaves of the seedlings (approximately 10 cm high) were collected and cut into 10-mm-long segments 35 mm below their apical tip. Forty segments were used for each treatment. The fresh weight of ten segments was measured with an analytical balance and placed in 25 × 90 mm (four vials per treatment) glass vials containing 10 mL of BAP (0, 0.5, 1, 2, 4 and 8 mg L^−1^), plant extracts or Agrostemin^®^, all of which were dissolved in deionized water. Deionized water was used as the negative control. The vials with cuttings were placed back in the dark growth chamber for 4 days. After 4 days of incubation, the cuttings were removed from the vials and transferred to test tubes containing 8 mL of 80% ethanol. The test tubes were transferred to a water bath (heated to 80–90 °C). After 10 min of chlorophyll extraction, the solution was cooled under running tap water and replenished to 10 mL with 80% ethanol. Evaporation was prevented by covering the test tubes. The cooled chlorophyll extract without the segments was then carefully poured into spectrophotometer cuvettes. The optical density (absorbance) was determined at 645 nm. The optical density was compared with 100 mg of fresh weight, and the adjusted results were compared with the activity of the different BAP concentrations. Each treatment was replicated four times, and the experiment was repeated three times.

### 4.3. Plant Hormone Analysis

Three freeze-dried, crushed samples of the plant materials and one dry sample of the biostimulant Agrostemin^®^ [10] were analyzed and quantified by liquid chromatography-mass spectrometry (LC-MS). Approximately 80–100 mg of the samples were extracted for hormone analysis (auxins: indole-3-acetic acid IAA, methyl-IAA, IAA-Ala, IAA-Trp and IAA-Asp; cytokinins: t-Zeatin (tZ), c-Zeatin (cZ), t-ZeatinRiboside (t-ZR) and c-ZeatinRiboside (cZR)). The samples were ionized for the high-resolution and precise quantification of the mass spectrometric detection. The data were normalized based on internal standards to account for experimental variation and hormone extraction/ionization efficiency. The samples were reconstituted in 200 μL of 15% methanol, and 10 μL were injected (1/10 of the sample). Plant hormone separation and quantification was performed by Creative Proteomics [26]. The amounts of the hormones were reported in ng g^−1^ of dry weight. The ‘Limit of Detection’ (LOD; with a 95% level of certainty) for the auxin detection was between 0.16 and 8 nM, and the LOD for the cytokinin detection was between 0.032 and 0.012 nM.

### 4.4. Statistical Analysis

The statistical package SPSS^®^ 29.0 (SPSS Inc., Chicago, IL, USA) was used for data analysis. The level of statistical significance *(p*) between the different treatments was determined using the Kruskal–Wallis test for the independent samples. Differences at *p* ≤ 0.05 were considered statistically significant. Each treatment was replicated four times, and the experiments were repeated three times.

## 5. Conclusions

*Agrostemma githago* (corn cockle) is an example of a “higher plant-derived biostimulant” (hPDB) that stimulates natural plant processes by controlling growth and development, as demonstrated when the water suspensions extracted from the seeds, seedlings and young plants exhibited auxin-like activity and low cytokinin-like activity. The LC-MS analysis of the dry plant materials confirmed that *A. githago* is a source of natural phytohormones. The preparations from this plant can be used in organic and sustainable agriculture at least for root induction. Using hPDBs can be an innovative strategy to improve plant growth, development and other quality traits of multiple plant species at different stages of development, with the ability to improve productivity and sustainability.

## Figures and Tables

**Table 1:** LC-MS analysis of auxines and their analogues in the dry plant materials of *Agrostemma githago* and Agrostemin^®^.

Sample	IAA	Methyl IAA	IAA-Ala	IAA-Asp	IAA-Trp
	ng g^−1^ of dry weight				
Agrostemin^®^	1.2	ND	ND	ND	ND
Seeds	3.3	ND	ND	28.0	0.4
Seedlings	53.4	ND	ND	7.9	ND
Young plants	15.9	ND	ND	ND	ND
LOD, nM 10 uL injection		0.16	8	0.4	0.4

Auxins: indole-3-acetic acid (IAA); methyl indole-3-acetic acid (methyl IAA), IAA-conjugates: indole acetyl-L-alanine (IAA-Ala), indole acetyl-L-aspartic acid (IAA-Asp), IAA-tryptophan (IAA-Trp); ND, not detected.

**Table 2:** The LC-MS analysis of the cytokinins and their analogues in the dry plant material of *Agrostemma githago* and Agrostemin^®^.

Sample	cZ	cZR	tZ	tZR
	ng g^−1^ of dry weight			
Agrostemin^®^	0.01	0.00	ND	ND
Seeds	0.15	0.11	0.21	0.06
Seedlings	0.09	1.88	0.52	2.09
Young plants	0.30	1.73	0.81	2.13
LOD, nM 10 uL injection			0.032	0.0128

Cytokinins: c-Zeatin (cZ) and t-Zeatin (tZ); cytokinin conjugates: c-ZeatinRiboside (c-ZR) and t-ZeatinRiboside (tZR); ND, not detected.

**Table 3:** The LC-MS analysis of the PGRs and their analogues cis-trans abscisic acid (ABA), salicylic acid (SA), jasmonic acid (JA), jasmonic acid-isoleucine (JA-Ile) and 12-oxo-phytodienoic acid (OPDA) in the dry plant material of *Agrostemma githago* and Agrostemin^®^.

Sample	ABA	SA	JA	JA-ILE	OPDA
	ng g^−1^ of dry weight				
Agrostemin^®^	0.5	129.4	ND	ND	ND
Seeds	5.3	157.0	ND	ND	5.9
Seedlings	33.4	193.4	65.6	22.0	22.3
Young plants	22.1	121.1	36.6	5.3	ND
LOD, nM 10 uL injection			0.3	0.1	8.0

Cis-trans abscisic acid (ABA), salicylic acid (SA), jasmonic acid (JA), jasmonic acid-isoleucine (JA-Ile) and 12-oxo-phytodienoic acid (OPDA).

## Data Availability

The data are available upon request from jana.ambrozic@um.si.
